# Effect of Solid-State Phase Transformation and Transverse Restraint on Residual Stress Distribution in Laser–Arc Hybrid Welding Joint of Q345 Steel

**DOI:** 10.3390/ma17112632

**Published:** 2024-05-29

**Authors:** Ruiyang Feng, Denggao Liu, Chaohua Zhang, Yunlong Pan, Yanjun Wang, Jie Chen, Xiaojun Ye, Min Lei, Yulong Li

**Affiliations:** 1 Key Lab for Robot and Welding Automation of Jiangxi Province, School of Advanced Manufacturing, Nanchang University, Nanchang 330031, China; 5904121057@email.ncu.edu.cn (R.F.); 412400230063@email.ncu.edu.cn (D.L.); yexiaojun0512@126.com (X.Y.); leimin@ncu.edu.cn (M.L.); 2 Railway Jiujiang Bridge Engn Co., Ltd., Jiujiang 332004, China; 18000289159@163.com; 3 Shanghai Institute of Optics and Fine Mechanics, Chinese Academy of Sciences, Shanghai 201800, China; wangyanjun686@126.com; 4 Institute of Special Equipment Inspection and Research, Jiangxi General Institute of Testing and Certification, Nanchang 330029, China; ncuchenjie@126.com

**Keywords:** high-strength low-alloy steel, laser–arc hybrid welding, solid-state phase transformation, transverse restraint, welding residual stress, numerical simulation

## Abstract

A Q345 steel butt-welded joint was manufactured using laser–arc hybrid welding (LAHW) technology, and its microstructure, microhardness, and residual stress (RS) distribution were measured. Using ABAQUS software, a sequentially coupled thermo-metallurgical-mechanical finite element method was employed to model the welding RS distribution in the LAHW joint made of Q345 steel. The effects of solid-state phase transformation (SSPT) and transverse restraint on the welding RS distribution were explored. The results show that a large number of martensite phase transformations occurred in the fusion zone and heat-affected zone of the LAHW joint. Furthermore, the SSPT had a significant effect on the magnitude and distribution of RS in the LAHW joint made of Q345 steel, which must be taken into account in numerical simulations. Transverse restraints markedly increased the transverse RS on the upper surface, with a comparatively minor impact on the longitudinal RS distribution. After the transverse restraint was released, both the longitudinal and transverse RS distributions in the LAHW joint reverted to a level akin to that of the welded joint under free conditions.

## 1. Introduction

High-strength low-alloy (HSLA) steels with medium thickness are widely used in steel structure fields, such as petrochemicals, shipbuilding, construction, and bridges. To improve the manufacturing efficiency, efficient and stable welding technology is very important for the manufacturing of medium-thickness steel plates. Laser–arc hybrid welding (LAHW) technology has broad prospects in the manufacture of medium-thickness steel plates, owing to advantages such as its fast welding speed, deep penetration, and high process stability [1,2,3]. During the welding process, welding residual stress (RS) is inevitably generated due to local rapid heating and cooling. In addition to welding thermal stress, the solid-state phase transformation (SSPT) of materials and boundary restraints of welded joints have important effects on the welding RS [4]. Excessive welding RS can result in diminished weld strength or even crack formation and adversely impact the overall performance of the workpiece [5]. Therefore, it is of great importance to study the influence of SSPT and boundary restraints on the RS distribution of LAHW joints made of HSLA steels with medium thickness.

Over the past decade, researchers have conducted investigations into the RS distribution of LAHW joints undergoing various hybrid welding processes. Sun et al. [6] studied the RS distribution in the LAHW joint made of NV E690 by combining numerical simulation and experiments, and they found that higher tensile RS distributes in the FZ and near the HAZ. Chen et al. [7] explored the influence of interactions between weld beads on the RS distribution in the multi-pass LAHW joint made of 316L stainless steel. Their research revealed that the peak value of the longitudinal RS appears near the fusion zone (FZ), while that of the transverse RS occurs near the heat-affected zone (HAZ). Kong et al. [8] studied the effect of welding speed on the RS distribution in the LAHW joint made of A36 mild steel and found that increased welding speed could reduce the welding RS. Ma et al. [9] explored the impact of different energy ratios between the arc and laser on the RS distribution in the LAHW joint made of SS400 steel. Their results showed that the distribution width of the high RS changes proportionally to the energy ratio, and the LAHW joint in the case of the largest energy ratio exhibits the highest RS. Xu et al. [10] investigated the influence of the number of welded passes on RS in the LAHW joint made of Q460 steel. They demonstrated that the transverse tensile RS at the weld root in multi-pass LAHW is much higher than that in single-pass LAHW. In addition to the influence of various hybrid welding processes on the RS distribution in LAHW joints made of HSLA steel, nonuniform microstructures and mechanical properties induced by SSPT in the FZ and HAZ could also affect the welding RS distribution [11]. Nitschke-Pagel and Wohlfahrt [12] discovered that the welding RS in low-alloy ferritic steels is significantly affected by SSPT at low phase-transformation temperatures using experimental measurements. Sun et al. [13] investigated the effect of SSPT on the RS distribution in the arc welded joint made of S355 steel. Their results indicated that SSPT has an impact on the RS distribution around the weld. Compared with arc welded joints, the cooling rates of LAHW joints are faster, and the phase types and proportions are very different. Variations in the microstructure and microhardness further lead to differences in the welding RS. However, there has been very little research conducted to clarify the influence of SSPT on the RS distribution in HSLA steel LAHW joints.

With the development of steel structures geared toward the large scale and highly parameterized, the boundary restraints of welded joints become more complex [14]. Sun et al. [15] explored the effect of restraint intensity on the welding RS distribution in H-Type cracking test specimens. Their research showed that the magnitude of the longitudinal RS could be influenced by the restraint intensity only in welded joints with shorter weld lengths. Venkatkumar and Ravindran [16] designed boundary restraints located at different distances from the welding bead, and found that the impact of boundary restraints on the transverse RS distribution of butt-welded joints is greater than that on their longitudinal RS distribution. Liu et al. [17] investigated the effect of saddle-shaped restraining plates on the RS distribution of butt-welded joints. Their findings indicated that the saddle-shaped restraining plates could increase the longitudinal RS in local welding regions where they are welded. Li et al. [18] studied the influence of stiffener dimension and location on the welding RS distribution in T-joints, and they demonstrated that structural restraint can significantly reduce the RS of flange surfaces and web facing the stiffener. Nonetheless, there has been limited investigation into the impact of transverse restraints on the RS distribution in LAHW joints.

In this study, the microstructure, microhardness, and RS distribution of the LAHW joint were measured through experimentation. The welding RS distribution was simulated using the sequentially coupled thermo-metallurgical-mechanical (TMM) finite element (FE) method. This investigation meticulously explored the effect of SSPT on the welding RS distributions and comprehensively compared the welding RS before and after the release of transverse restraints.

## 2. Experimental Procedure

**Figure 1:**
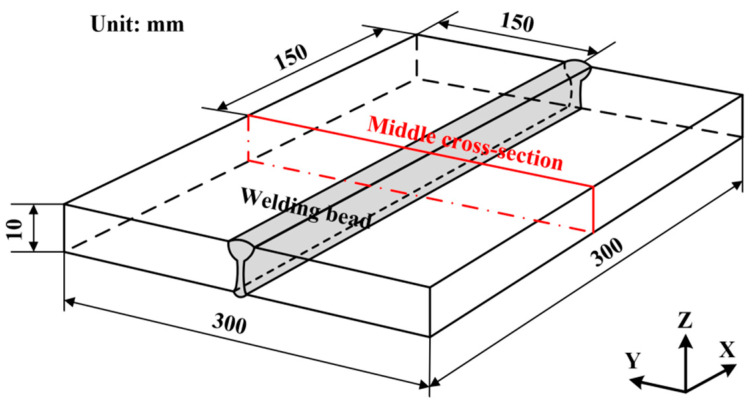
Geometric dimensions of the specimen.

In this work, the base metal was Q345 steel, while the filler material was ER50-2 wire. The chemical compositions (mass fraction, %) of both the Q345 steel and ER50-2 wire are presented in Table 1. A butt-welded joint made of 10 mm thick plates was fabricated using LAHW technology, and its geometric dimensions are shown in Figure 1. The welding parameters are shown in Table 2. During the welding process, the specimen was under no external restraints that limited its movement or rotation.

After welding, the RS distributions on the upper and bottom surfaces of the LAHW joint were measured by using the hole-drilling strain method [19]. A macro specimen of the LAHW joint was obtained through wire cut electrical discharge machining. After grinding, polishing, and etching with 4% Nital solution, the microstructure of the LAHW joint was identified with a Leica DM4M microscope (Leica, Wetzlar, Germany). The microhardness of the LAHW joint was measured using a TMHV-1000Z Vickers microhardness tester (Tuming Intelligent, Changzhou, China).

## 3. Numerical Analysis

**Figure 2:**
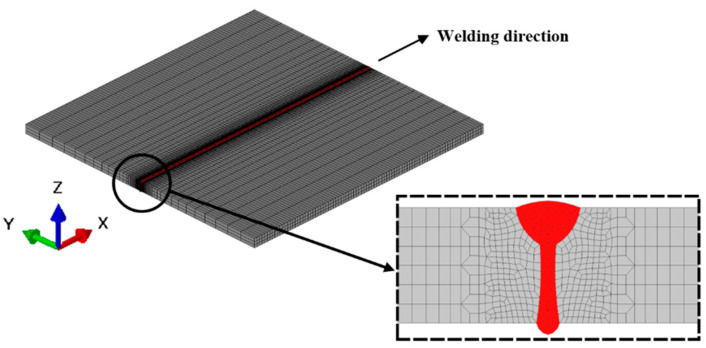
FE model of the LAHW joint.

**Figure 3:**
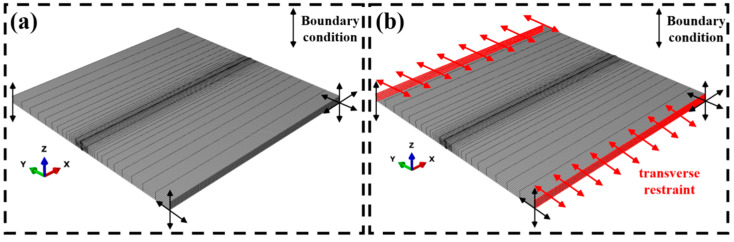
Boundary conditions of the FE model under: (**a**) free condition; (**b**) transverse restraint condition.

A sequentially coupled TMM FE method was developed to simulate the temperature field and stress–strain field of the LAHW joint. Figure 2 illustrates the FE model with the exact size of the actual LAHW joint. To achieve an equilibrium between calculation time and accuracy, sparse mesh was positioned in regions distant from the FZ, whereas dense mesh was placed in the FZ and its vicinity. The FE model comprised a total of 42,884 elements and 53,756 nodes. The element types employed in the temperature field and stress–strain field were DC3D8 and C3D8I, respectively [18]. To prevent rigid body movement of the FE model, boundary conditions of six degrees of freedom were applied to the three boundary nodes of the model, as shown in Figure 3a. To study the effect of boundary restraints on the RS distribution of the LAHW joint, the transverse restraint was imposed on the FE model in the numerical simulation, as shown in Figure 3b. The Y-direction shrinkage of the LAHW joint under transverse restraint condition was limited.

### 3.1. Thermal Analysis

Heat conduction inside the workpiece followed Fourier’s law. The nonlinear heat transfer equation [20] is as follows:

(1) $$\rho c\frac{\partial T}{\partial t}=\frac{\partial }{\partial x}\left[\lambda_{x}\frac{\partial T}{\partial x}\right]+\frac{\partial }{\partial y}\left[\lambda_{y}\frac{\partial T}{\partial y}\right]+\frac{\partial }{\partial z}\left[\lambda_{z}\frac{\partial T}{\partial_{z}}\right]+Q$$

where *ρ* stands for the density; *c* represents the specific heat capacity; *λ* denotes the thermal conductivity; *T* represents the instantaneous temperature at the nodes of the FE model; *t* is the welding time; and *Q* stands for the internal heat source intensity.

For the temperature field analysis of the LAHW joint, selecting right heat source models in the numerical simulation is essential. The double ellipsoid heat source model [21] was selected to imitate the welding heat input during the arc welding process, while the three-dimensional Gaussian cone heat source model [22] was selected to imitate the welding heat input during the laser welding process. These two heat source models were integrated to establish a hybrid heat source model to simulate the welding heat input on the LAHW joint, which was accomplished by employing the user subroutine DFLUX in the ABAQUS software (https://www.3ds.com/products/simulia/abaqus, accessed on 1 May 2024).

The front quadrant (*q_f_*) and rear quadrant (*q_r_*) of the heat flux within the double ellipsoid heat source are expressed by the following equations.

(2) $$q_{f}(x,y,z,t)=\frac{6\sqrt{3}f_{f}Q_{1}}{\pi \sqrt{\pi }a_{f}bc}e^{-3\left[\frac{{\left(x-x_{0}-vt\right)}^{2}}{{a_{f}}^{2}}+\frac{{\left(y-y_{0}\right)}^{2}}{b^{2}}+\frac{{\left(z{-z}_{0}\right)}^{2}}{c^{2}}\right]}$$

(3) $$q_{r}(x,y,z,t)=\frac{6\sqrt{3}f_{r}Q_{1}}{\pi \sqrt{\pi }a_{r}bc}e^{-3\left[\frac{{\left(x-x_{0}-vt\right)}^{2}}{{a_{r}}^{2}}+\frac{{\left(y-y_{0}\right)}^{2}}{b^{2}}+\frac{{\left(z{-z}_{0}\right)}^{2}}{c^{2}}\right]}$$

(4) $$Q_{1}=\eta_{1}UI$$

where *x*_0_, *y*_0_, and *z*_0_ are the double ellipsoid heat source model’s center position coordinates; *a_f_*, *a_r_*, *b*, and *c* stand for the double ellipsoid heat source model’s semi-axis; *Q*_1_ denotes the arc welding power; and *f_f_* and *f_r_* stand for the distribution parameters of the heat flux for the front and rear quadrants in the double ellipsoid heat source model. In this study’s thermal analysis, *f_f_* = 0.6 and *f_r_* = 1.4; *v* denotes the welding speed; *I* represents the welding current; *U* denotes the arc voltage; and *η*_1_ stands for the efficiency of the arc welding.

The heat flux within the 3D Gaussian cone heat source model can be described by the following equations:

(5) $$q(r,z)=\frac{9\eta_{2}Q_{2}e^{3}}{\pi H(e^{3}-1)(r_{e}^{2}+r_{e}r_{i}+r_{i}^{2})}\exp(-\frac{3r^{2}}{r_{0}^{2}})$$

(6) $$r_{0}=r_{e}-\frac{\left(r_{e}-r_{i})(z_{e}-z\right)}{z_{e}-z_{i}}$$

(7) $$H=z_{e}-z_{i}$$

where *r_e_* stands for the radius of the upper surface, while *r_i_* represents the radius of the bottom surface; *z_e_* and *z_i_* represent the Z-coordinates of the upper and bottom surfaces, respectively; *Q*_2_ denotes the laser welding power; and *η*_2_ stands for the efficiency of the laser welding. Table 3 shows the parameters for cone–double ellipsoid hybrid heat source model.

The convective heat dissipation followed Newton’s law, which is expressed as follows:

(8) $$q_{c}=-h_{c}\left(T_{s}-T_{0}\right)$$

where *h_c_* stands for the heat transfer coefficient; *T_s_* represents the surface temperature of the workpiece; and *T*_0_ denotes the ambient temperature, taken as 20 °C.

The radiative heat dissipation followed Boltzmann’s law, which is expressed as follows:

(9) $$q_{r}=-\varepsilon \sigma \left[{\left(T_{s}+273\right)}^{4}-{\left(T_{0}+273\right)}^{4}\right]$$

where *σ* denotes the Stefan–Boltzmann constant, and *ε* is the emissivity, which takes a value of 0.8.

**Figure 4:**
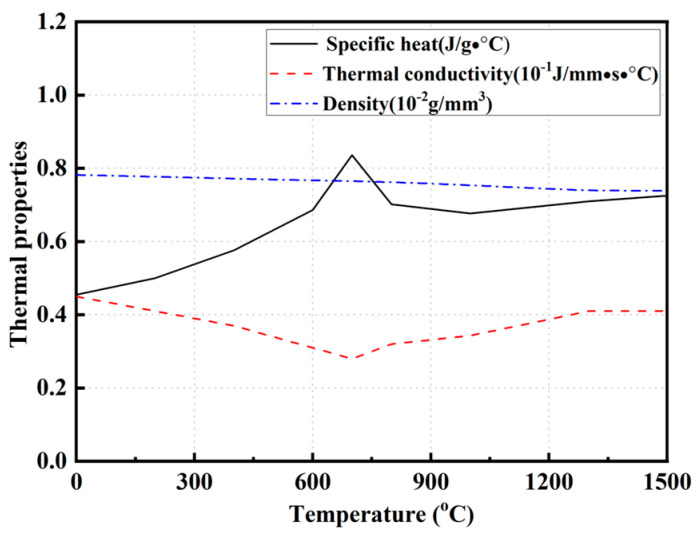
Parameters of high temperature thermal physical properties (from Ref. [23]).

During the temperature field calculation, it is assumed that the thermophysical property parameters of the weld metal and base metal are the same. Figure 4 [23] illustrates the temperature-dependent thermal physical property parameters of Q345 steel, including latent heat, thermal conductivity, and density. Convective and radiative heat dissipations were accomplished by employing the user subroutine FILM in the ABAQUS software.

### 3.2. Metallurgical Analysis

During the welding process, SSPT was simulated on the basis of the metallurgical analysis of the phase transformation laws of HSLA steel. The A1 and A3 temperatures of Q345 steel were calculated to be 708 °C and 826 °C, respectively, based on the empirical equations [13]. During the welding heating process, the base metal completely undergoes austenitization when the material temperature rises above A3. The base metal undergoes partial austenitization when the material temperature ranges between A1 and A3. A linear relationship was employed to simulate the process mentioned above. The increment in the austenite volume fraction (Δ*f_a_*) in the partially austenitized region can be calculated using the following equation [24]:

(10) $$\Delta f_{a}=\frac{\Delta T}{A_{3}-A_{1}}\times 100\%$$

where Δ*T* stands for the current temperature increment.

**Figure 5:**
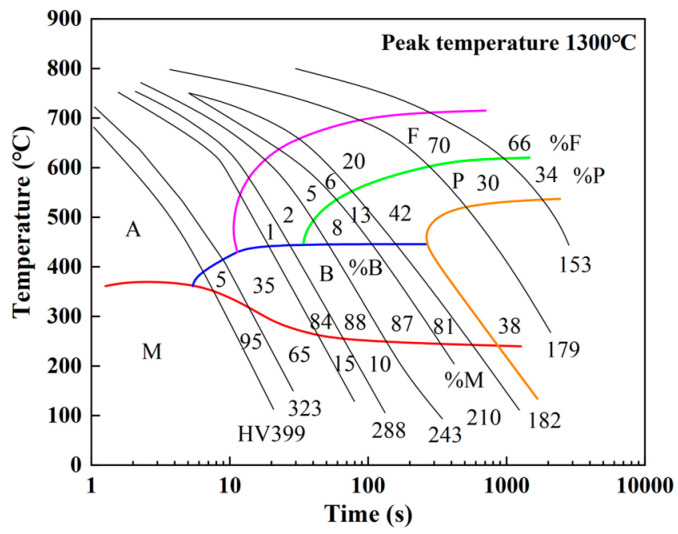
SH-CCT diagram of Q345 steel (from Ref. [25]).

During the cooling stage after welding, supercooled austenite starts to become unstable when the temperature drops below A3, and further cooling results in phase transformation. Figure 5 [25] illustrates the SH-CCT diagram of Q345 steel. The supercooled austenite mainly undergoes ferrite and pearlite phase transformations at lower cooling rates. While at higher cooling rates, it primarily undergoes bainite and martensite phase transformations.

Bainite, ferrite, and pearlite phase transformations are diffusive phase transformations, while martensite phase transformation is nondiffusive. The Johnson–Mehl–Avrami–Kolmogorov equation [26] is employed to elucidate the diffusive phase transformation process. The ferrite, pearlite, and bainite phase transformations are described by the following equations:

(11) $$f_{F}=1-\exp\left(-k_{F}t^{n_{F}}\right)$$

(12) $$f_{P}=\left(1-f_{F}\right)\left[1-\exp\left(-k_{P}t^{n_{P}}\right)\right]$$

(13) $$f_{B}=\left(1-f_{F}-f_{P}\right)\left[1-\exp\left(-k_{B}t^{n_{B}}\right)\right]$$

where *f_F_*, *f_P_*, and *f_B_* are volume fractions of ferrite, pearlite and bainite, respectively; *t* represents the time (s); and *k_i_* (*i* = *F*, *P*, *B*) and *n_i_* (*i* = *F*, *P*, *B*) are dynamic parameters related to the chemical composition, austenite grain size, and temperature. Based on the SH-CCT diagram of Q345 steel, these parameters are determined by using Scheul’s superposition rule [13].

The K–M relationship [27] is employed to elucidate the nondiffusive phase transformation process. The martensite phase transformation is described by the following equation:

(14) $$f_{M}=\left(1-f_{F}-f_{P}-f_{B}\right)\left\{1-\exp\left[-b\left(M_{s}-T\right)\right]\right\}\left(T\leq M_{s}\right)$$

where *f_M_* is the martensite volume fraction; *M_s_* stands for the temperature when the martensite phase transformation begins; *T* represents the current temperature in the cooling process; and *b* stands for martensite phase transformation rate, which is a constant associated with the material. For HSLA steels, the value of *b* is 0.011 [24]. According to the SH-CCT diagram of Q345 steel, the starting temperature (*M_s_*) and ending temperature (*M_f_*) of the martensite phase transformation are 370 °C and 200 °C, respectively. The thermo-metallurgical phase transformation process was realized by employing the user subroutines HETVAL and SDVINI in the ABAQUS software.

### 3.3. Mechanical Analysis

**Figure 6:**
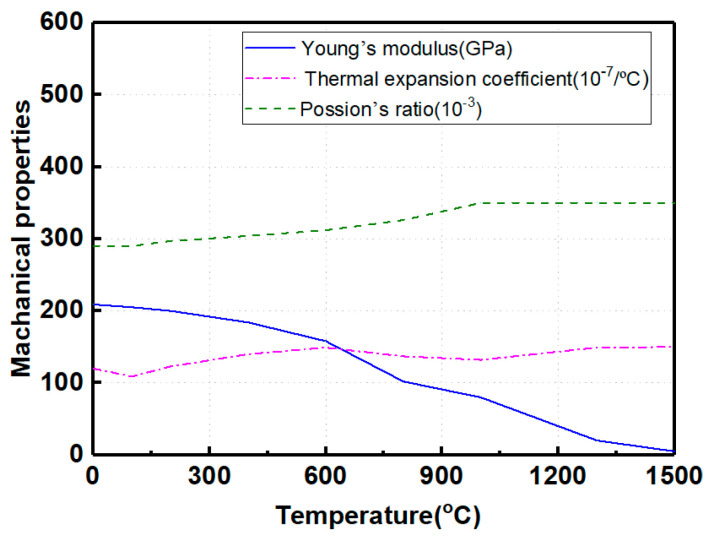
Temperature-dependent mechanical properties (from Ref. [23]).

In the calculation of the stress–strain field, the same FE mesh model as the temperature field calculation was used. The results of the temperature field calculations at each node of the FE model were loaded into the stress–strain field calculation as the heat load. The influence of transformation-induced plasticity (TRIP) on welding RS caused by SSPT in Q345 steel remains unclear and lacks relevant parameters for TRIP. Consequently, the stress–strain field calculation did not account for the TRIP strain of Q345 steel. However, the volume changes and mechanical property parameter changes in the material induced by SSPT were considered. Q345 HSLA steel dissipates heat quickly during the welding process, resulting in a short residence time at high temperatures. The creep process has little effect on the welding RS, so the stress–strain field calculation did not take the creep process of Q345 steel into account. The expression for the total strain at each material point is

(15) $$\varepsilon_{total}=\varepsilon_{e}+\varepsilon_{p}+\varepsilon_{th}$$

where *ε_e_* stands for elastic strain; *ε_p_* represents plastic strain; and *ε_th_* denotes thermal and metallurgical strain. In the stress–strain field calculation, *ε_e_* was calculated according to the isotropic Hooke’s law, and *ε_p_* was calculated according to the Mises yield criterion. Figure 6 [23] illustrates the variations in the Poisson’s ratio, thermal expansion coefficient, and Young’s modulus of Q345 steel with temperature.

**Figure 7:**
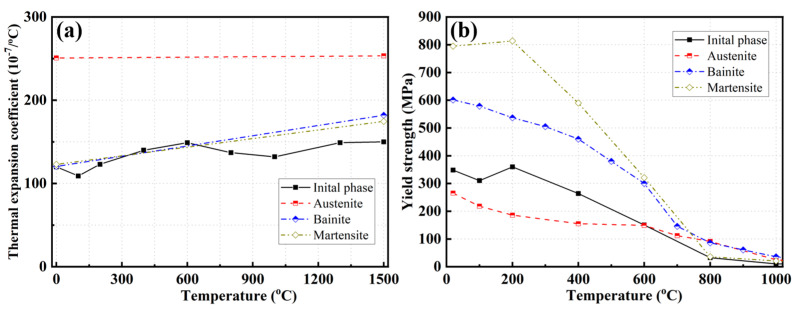
Thermal expansion coefficients and yield strength of each phase (from Ref. [13]): (**a**) thermal expansion coefficient; (**b**) yield strength.

The user subroutine UEXPAN in the ABAQUS software was employed to numerically simulate the volume change in the phases due to SSPT. Figure 7 [13] shows the thermal expansion coefficients and yield strengths corresponding to the various phases of Q345 steel.

### 3.4. Simulation Cases

In Table 4, three cases were designed to explore the effects of SSPT and transverse restraint on the RS distribution in the Q345 steel LAHW joint. In Case 1, SSPT was ignored in the numerical simulation, and the FE model was under free conditions. Based on Case 1, Case 2 incorporated the consideration of SSPT into the material model to investigate the influence of SSPT on the welding RS. Compared with Case 2, the material model in Case 3 was identical, but the transverse restraint was imposed on the FE model to study its effect on the welding RS.

## 4. Results and Discussion

### 4.1. Thermal and Metallurgical Results Analysis

**Figure 8:**
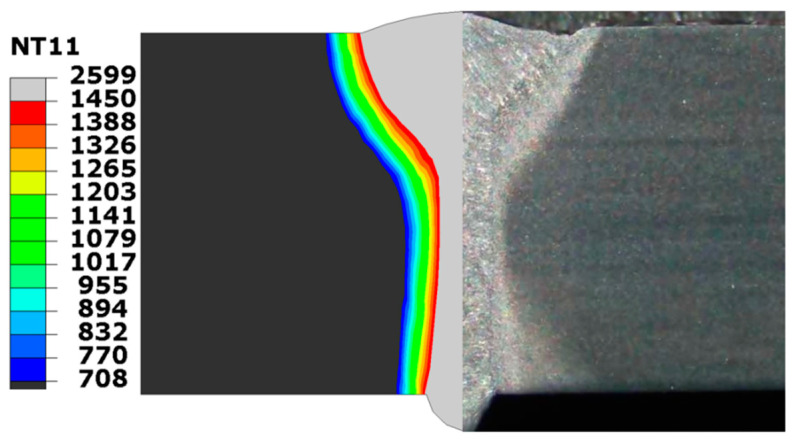
Peak temperature (°C) distribution in the middle cross-section.

Figure 8 illustrates the peak temperature distribution and macromorphology of the middle cross-section in the LAHW joint. The FZ is the gray region characterized by peak temperatures exceeding 1450 °C, while the HAZ is the area where temperatures peak below 1450 °C but remain above 708 °C. The sizes and shapes of the FZ and HAZ simulated using the FE method match those observed in the actual LAHW joint very well. The appropriateness of using the established cone–double ellipsoid hybrid heat source model to simulate the temperature field of the LAHW joint is verified.

**Figure 9:**
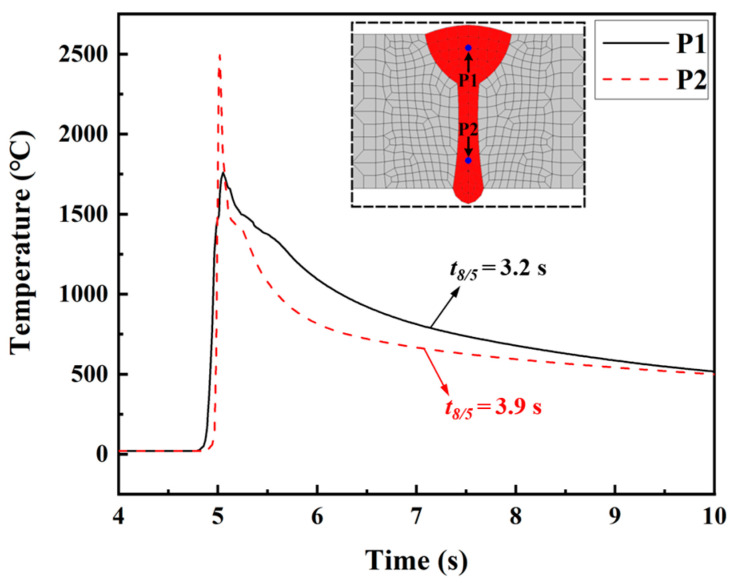
Temperature histories at points P1 and P2.

**Figure 10:**
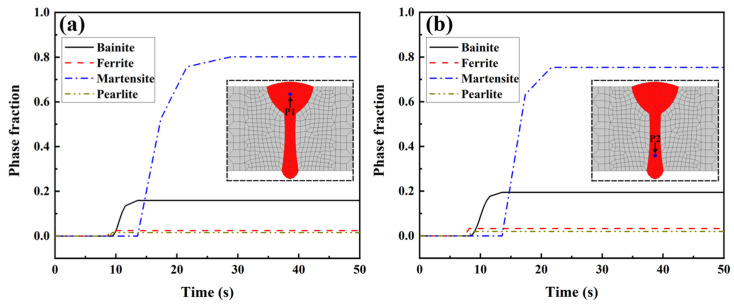
The calculated phase fractions: (**a**) point P1; (**b**) point P2.

Figure 9 illustrates the historical temperature data obtained from the numerical simulation at points P1 and P2. Point P1 is located in the FZ of the arc zone in the LAHW joint, while point P2 is situated in the FZ of the laser zone. Compared with the temperature history at point P1, the peak temperature at point P2 was higher, and the cooling rate in the high-temperature stage was faster. But the *t*_8/5_ values at points P1 and P2 were similar. This result is mainly because the welding heat input from laser welding is more concentrated than that of arc welding. Therefore, the cooling rate in the laser welding area was faster than that in the arc welding area at high temperatures. As the temperature decreased, the temperature gradient of the LAHW joint slowed down, and the *t*_8/5_ values at points P1 and P2 were relatively close. Figure 10 displays the calculated phase fractions at points P1 and P2. The figure shows that the martensite content in the FZ was the highest, nearing 0.8, followed by the bainite content, which was around 0.2. The ferrite and pearlite contents were very low. The main reasons for these phenomena are the low heat input and fast cooling rate of the LAHW joint.

**Figure 11:**
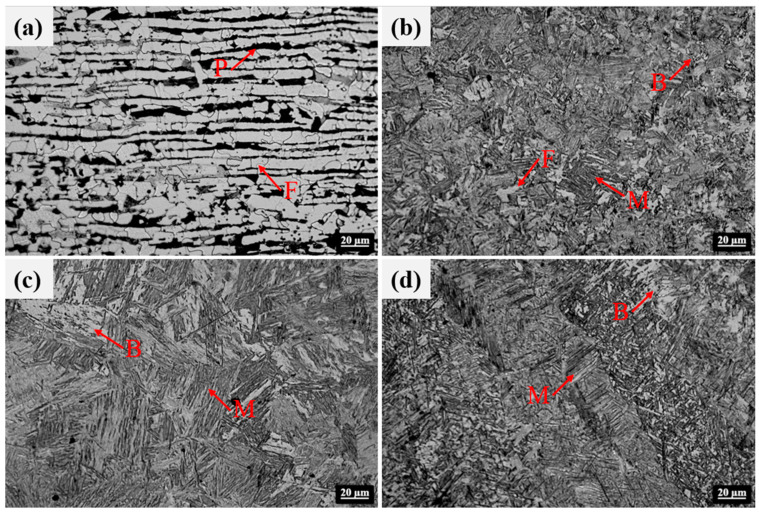
Microstructures of the LAHW joint: (**a**) base metal; (**b**) fine grain zone; (**c**) coarse grain zone; (**d**) FZ.

**Figure 12:**
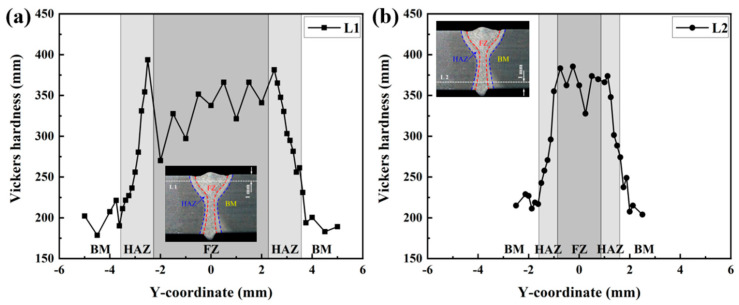
Hardness distributions in the LAHW joint: (**a**) L1; (**b**) L2.

Figure 11 displays the microstructure of each region in the LAHW joint. The microstructure in the base metal was mainly composed of ferrite and pearlite. The fine grain zone primarily consisted of martensite, granular bainite, and ferrite. The microstructure in the coarse grain zone was predominantly composed of lamellar martensite, with a small amount of bainite. Martensite and black acicular bainite can be observed in the FZ. Figure 12 illustrates the hardness distributions at a distance of 1 mm from the upper and bottom surfaces of the LAHW joint. The figure shows that the hardness of the FZ in the LAHW joint was in the high hardness range, with a peak hardness of 393.6 HV. The SH-CCT diagram of Q345 steel in Figure 5 indicates that the microstructure with a hardness of 393.6 HV mainly consisted of martensite, with a minor bainite presence. This fact is mainly due to the small heat input and fast cooling rate of LAHW, resulting in many martensite microstructures in the FZ. The calculated phase fraction in Figure 10 corresponds to the microstructure and microhardness of the actual LAHW joint in Figure 11 and Figure 12, indicating the rationality of the calculated temperature field and the established SSPT model in the numerical simulation.

### 4.2. Mechanical Result Analysis

#### 4.2.1. Effect of SSPT on Welding RS

**Figure 13:**
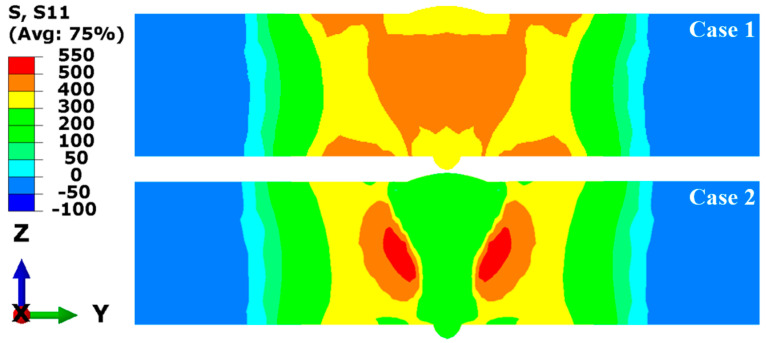
Longitudinal RS (MPa) distributions of the middle cross-section in Case 1 and Case 2.

Figure 13 compares the longitudinal RS distributions in the middle cross-section simulated in Case 1 and Case 2. The contour results reveal that there were significant differences in the magnitudes and distributions of the RS within and near the FZ and HAZ between Case 1 and Case 2, while the differences are minimal in the areas far from the FZ. In Case 1, the high tensile RS occurred in and near the FZ and HAZ, and the tensile RS was balanced by the compressive RS located away from the FZ. The peak value of the tensile RS exceeds the yield strength of the base metal at room temperature (345 MPa), up to 500 MPa. In Case 2, it can be observed that the longitudinal RS in the FZ and HAZ was in low tensile stress state, while high tensile RS occurred in the middle area of the plate’s thickness near the HAZ. This suggests that SSPT can significantly reduce the predictive values of the longitudinal RS in the FZ and HAZ for HSLA steel LAHW joints. The main reason for this phenomenon is that martensite and bainite phase transformations primarily occur in the FZ and HAZ of the LAHW joint. During the welding cooling process, the transformation of supercooled austenite into martensite and bainite precipitates volumetric expansion. Subsequently, the tensile plastic strains engendered by the cooling process are compensated, resulting in a decrease in the high tensile RS in the FZ and HAZ of the LAHW joint.

**Figure 14:**
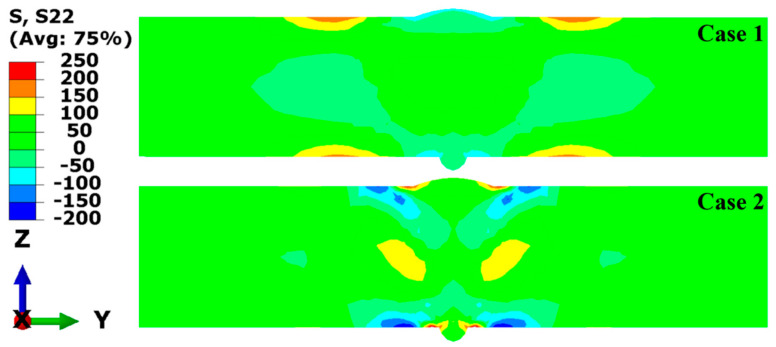
Transverse RS (MPa) distributions of the middle cross-section in Case 1 and Case 2.

Figure 14 illustrates the transverse RS distributions of the middle cross-section in Case 1 and Case 2. The plotted results show that the transverse RS distributions in the two cases were significantly different in the FZ and the area away from the FZ. In Case 1, the high tensile RS occurred on the upper and bottom surfaces away from the FZ, and the RS in other areas exhibited low tensile stress or low compressive stress. Observing Case 2, the high tensile RS can be found at the weld toe of the upper and bottom surfaces and in the middle area of the plate’s thickness near the HAZ, while the high compressive RS is distributed on the upper and bottom surfaces near the HAZ. This suggests that SSPT not only has an important impact on the transverse RS distribution in and near the FZ but also reduces the high tensile RS on the upper and bottom surfaces away from the FZ.

**Figure 15:**
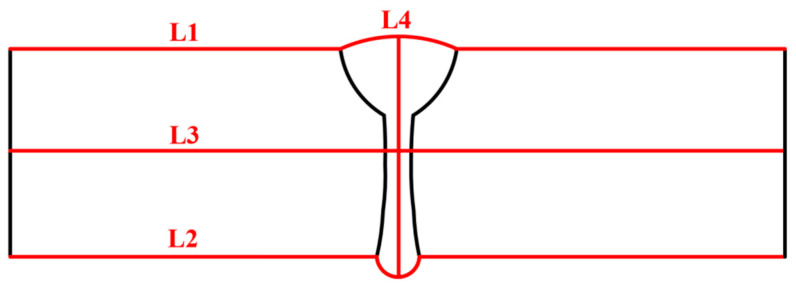
Path schematic in middle cross-section of the LAHW joint.

**Figure 16:**
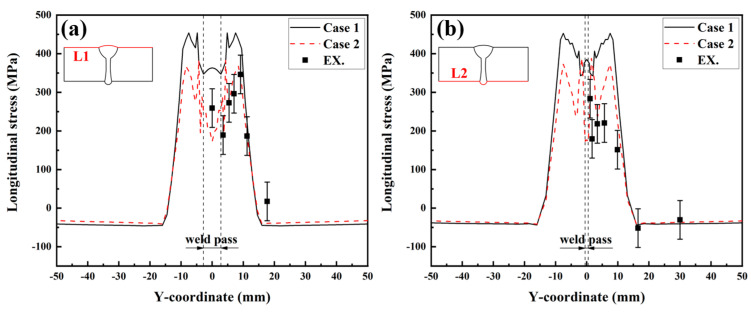
Longitudinal welding RS distributions: (**a**) L1; (**b**) L2.

To quantitatively study the effect of SSPT on the welding RS distribution, the RS distributions along Paths L1, L2, L3, and L4 were compared. The path schematic in the middle cross-section of the LAHW joint is shown in Figure 15. Figure 16a shows the simulated and experimentally measured longitudinal RS distributions along Path L1. The experimental error of the hole-drilling strain method was approximately ±50 MPa [28]. In Case 1, the high tensile RS occurred in and near the FZ and HAZ. As the distance from the FZ and HAZ increased, the tensile RS decreased rapidly in the ranges of −8 mm < Y < −16 mm and 8 mm < Y < 16 mm, eventually transitioning into compressive RS. Comparing Case 1 and Case 2, it is evident that the overall tensile RS in and near the FZ and HAZ in Case 2 was significantly lower than that in Case 1. The peak value of the longitudinal RS in Case 1 was 454.2 MPa while that in Case 2 was 382.9 MPa. The SSPT can reduce the peak value of the longitudinal RS on the upper surface by 15.7%. The magnitude and distribution of the longitudinal RS away from the FZ in Case 2 remain nearly identical to those in Case 1. Comparing the experimental values and simulated values, it can be found that the longitudinal RS simulated in Case 2 more closely approximated the experimental measurement than that simulated in Case 1. This result indicates that the material model that does not consider SSPT will grossly overestimate the longitudinal RS of Q345 steel LAHW joints. The longitudinal RS distributions along Path L2 predicted by Case 1 and Case 2 are shown in Figure 16b. The overall “low–high–low” distribution trend in the longitudinal RS on the bottom surface was very similar to that on the upper surface. The peak values of the longitudinal RS for Case 1 and Case 2 were 453.1 MPa and 387.8 MPa, respectively. The SSPT can reduce the peak value of the longitudinal RS on the bottom surface by 14.4%.

**Figure 17:**
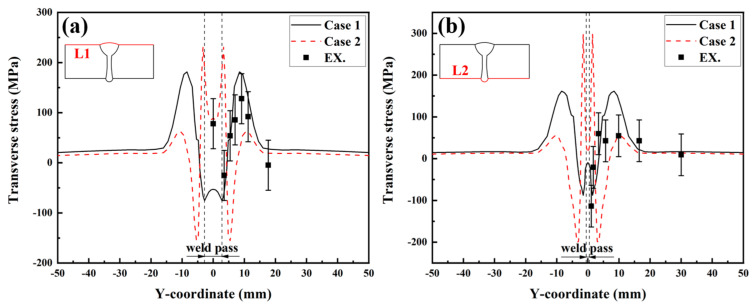
Transverse welding RS distributions: (**a**) L1; (**b**) L2.

Figure 17 displays the transverse RS distributions along Paths L1 and L2 simulated by the FE method with the corresponding experimental measurements. From Figure 17a, it can be seen that the transverse RS profile of Case 1 was M-shaped but that of Case 2 was W-shaped. A comparison between Case 1 and Case 2 reveals that the transverse RS distributions in these two cases were obviously different, in the range of −15 mm < Y < 15 mm. The transverse RS in the ranges of Y < −15 mm and Y > 15 mm were almost identical and near 0 MPa. By observing Figure 17b, it becomes evident that the distribution trends in the transverse RS in both Case 1 and Case 2 closely resemble those observed in Figure 17a. Comparing the experimental values and simulated values in Figure 17a,b, it can be found that the transverse RS simulated by Case 2 was in better agreement with the experimental measurements.

**Figure 18:**
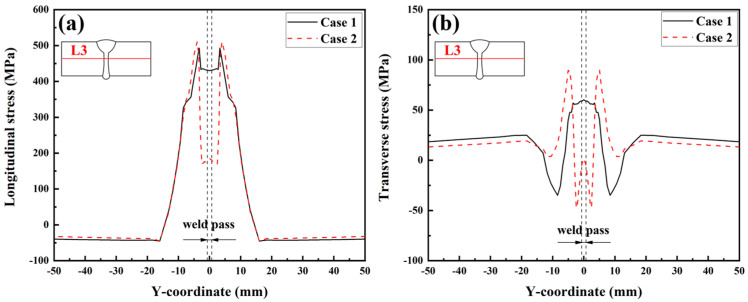
Welding RS distributions along L3: (**a**) longitudinal RS; (**b**) transverse RS.

Figure 18 presents the longitudinal and transverse RS distributions along Path L3 simulated in Case 1 and Case 2. Figure 18a illustrates that the longitudinal RS in the FZ and HAZ in Case 2 is significantly lower than that in Case 1. The peak value of the longitudinal RS in the FZ and HAZ in Case 1 and Case 2 are 436.3 MPa and 184.1 MPa, respectively. Considering SSPT into the material model can reduce the peak value of the longitudinal RS in the FZ and HAZ at the middle of the plate’s thickness by 57.8%. Further observation shows that the longitudinal RS in the base metal near the HAZ in Case 2 is higher than that in Case 1. This phenomenon occurs mainly because the expansion of the FZ and HAZ induced by the SSPT increases the restraint intensity of the base metal near the HAZ [29]. Figure 18b illustrates that the transverse RS simulated in Case 2 was lower than that simulated in Case 1, in the range of −3.6 mm < Y < 3.6 mm. The peak value of the transverse RS in the FZ in Case 1 was 60.2 MPa, while that in Case 2 was 1.9 MPa. In the ranges of −15 mm < Y < −3.6 mm and 3.6 mm < Y < 15 mm, the transverse RS in Case 2 was higher than that in Case 1. The transverse RS values modeled in these two cases were similar, in the ranges of Y < −15 mm and Y > 15 mm.

**Figure 19:**
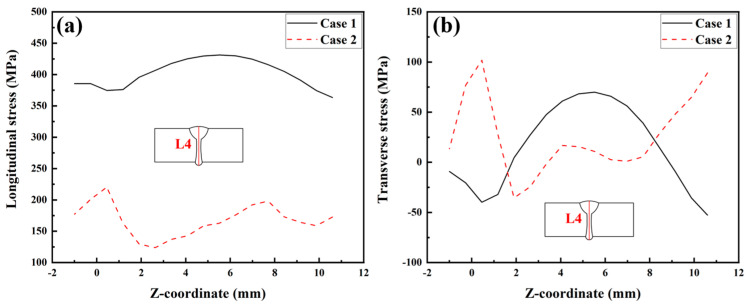
Welding RS distributions along L4: (**a**) longitudinal RS; (**b**) transverse RS.

Figure 19 shows the longitudinal and transverse RS distributions along Path L4. By observing Figure 19a, the average value of the longitudinal RS in Case 1 was 363.3 MPa while that in Case 2 was 172.7 MPa. This information suggests that SSPT can reduce the average value of the longitudinal RS in the FZ by 52.5%. According to Figure 19b, SSPT increases the transverse RS in the FZ near the upper and bottom surfaces. In the middle area of the plate’s thickness (in the range of 1.6 mm < Y < 8.2 mm) in the FZ, the SSPT can reduce the transverse RS. From Figure 19a,b, it can be concluded that the SSPT significantly reduced the longitudinal RS in the FZ of the LAHW joint and that it had a more complex impact on the transverse RS distribution. This fact is mainly because the transverse RS distribution is not only affected by the SSPT in the FZ and HAZ but also by changes in the longitudinal RS caused by the SSPT [30].

#### 4.2.2. Effect of Transverse Restraint on Welding RS

**Figure 20:**
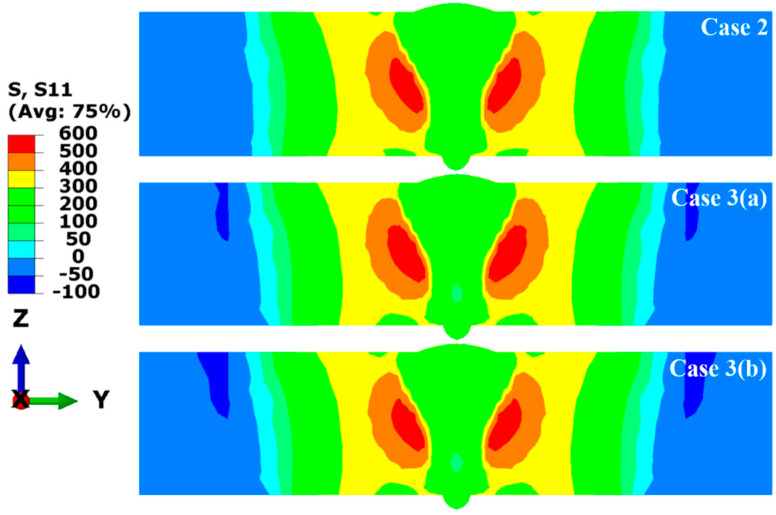
Longitudinal RS (MPa) distributions of the middle cross-section in Case 2, Case 3 (a) and Case 3 (b).

To study the effect of transverse restraint on the welding RS distribution in the LAHW joint, the RS distributions in the welded joint before (Case 3 (a)) and after (Case 3 (b)) releasing the transverse restraint were compared with the RS distribution of the welded joint under free conditions (Case 2), and Figure 20 demonstrates the longitudinal RS distribution in the middle cross-section simulated in Case 2, Case 3 (a), and Case 3 (b). The contour results indicate that the magnitude and distribution of the longitudinal RS simulated by these three cases are very similar. Case 2 reveals that the high tensile RS occurred in the middle area of the plate’s thickness near the HAZ, and the tensile RS in the FZ and HAZ was at a low level. The tensile RS was balanced by the compressive RS far from the FZ. Comparing with Case 2, it can be found that the tensile RS contours of Case 3 (a) and Case 3 (b) are almost identical. There were only slight differences in the compressive RS away from the FZ. A comparison of the obtained results among these three cases indicates that transverse restraints had little effect on the longitudinal RS distribution in the LAHW joints. The main reason for this phenomenon is that the longitudinal restraint intensity of LAHW joints is much stronger than the transverse restraint intensity, and the transverse restraint has a slight impact on the longitudinal restraint intensity [18].

**Figure 21:**
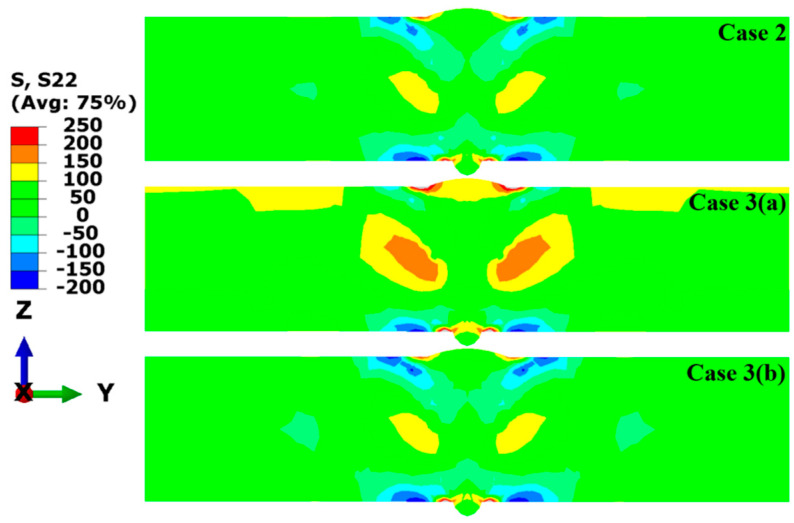
Transverse RS (MPa) distributions in the middle cross-section in Case 2, Case 3 (a) and Case 3 (b).

The transverse RS distribution contours of the middle cross-section simulated in Case 2, Case 3 (a), and Case 3 (b) are shown in Figure 21. Compared with Case 2, the transverse RS of the upper surface and middle area of the plate’s thickness near the HAZ simulated in Case 3 (a) increased significantly, while the transverse RS on the bottom surface increased slightly. This phenomenon occurs chiefly because transverse restraints limit the Y-direction shrinkage of LAHW joints during the welding process, resulting in a significant increase in the transverse tensile RS. Case 3 (b) shows that the transverse RS distribution of the LAHW joint changes considerably after releasing the transverse restraint. The magnitude and distribution of the transverse RS simulated by Case 3 (b) are very close to those simulated by Case 2. This fact suggests that, after releasing the transverse restraint, the transverse RS of the LAHW joint returns to a level comparable to that of the welded joint under free condition. Comparing Figure 20 and Figure 21, it can be found that the influence of the transverse restraint on the transverse RS distribution of the LAHW joint is much greater than that on the longitudinal RS distribution.

**Figure 22:**
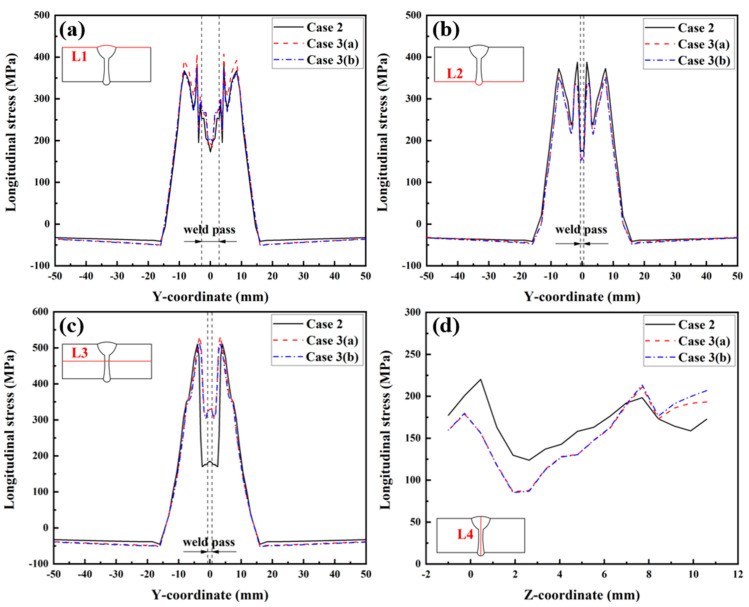
Longitudinal RS distributions in the LAHW joint: (**a**) L1; (**b**) L2; (**c**) L3; (**d**) L4.

The longitudinal RS distributions along Paths L1, L2, L3, and L4 are presented in Figure 22. From Figure 22a, it can be found that the magnitude and distribution of the longitudinal RS in the three cases were very similar, except for the longitudinal RS in the range of −9 mm < Y < 9 mm, at which the longitudinal RS in Case 3 (a) was slightly higher than that in the other two cases. Similar overall “low–high–low” distribution trends of the longitudinal RS can also be found along Paths L2 and L3, as shown in Figure 22b,c, and the longitudinal RS distributions among these three cases were almost the same, except for some slight differences in magnitude. Figure 22d reveals that the longitudinal RS along Path L4, as simulated in Case 3 (a) and Case 3 (b), was lower than that simulated in Case 2 in the range of Z < 7 mm, while it is higher in the range of Z > 7 mm.

**Figure 23:**
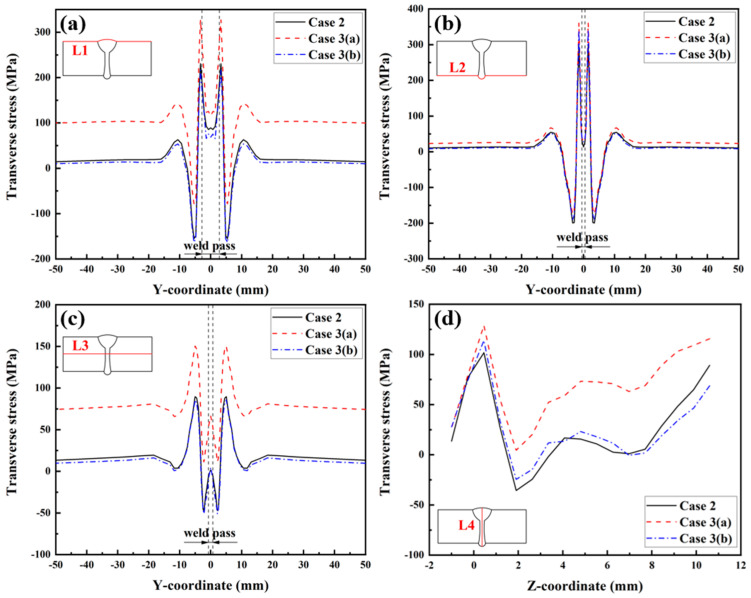
Transverse RS distributions in the LAHW joint: (**a**) L1; (**b**) L2; (**c**) L3; (**d**) L4.

**Figure 24:**
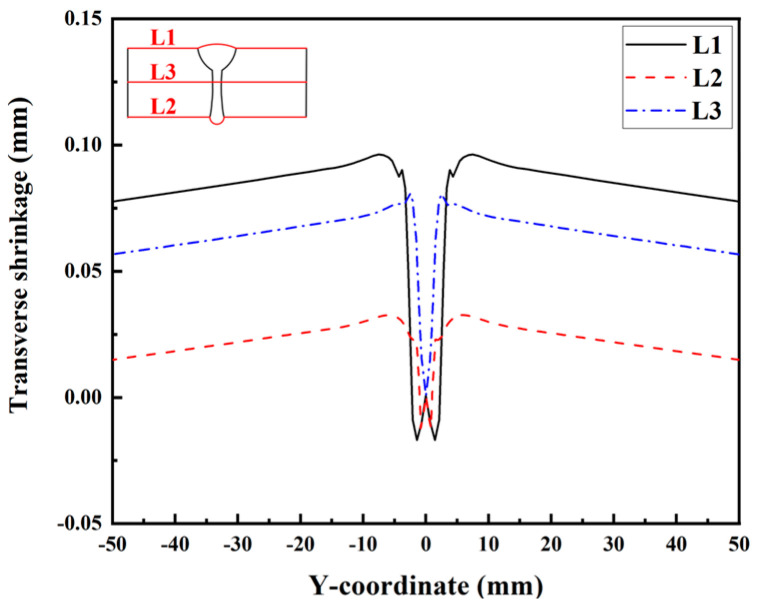
Transverse shrinkage deformations along L1, L2, and L3 in Case 2.

Figure 23 illustrates the transverse RS distributions along Paths L1, L2, L3, and L4. Observing Figure 23a,c, it is evident that the transverse RS simulated by Case 3 (a) is obviously higher than that simulated by Case 2. This information indicates that the transverse restraint significantly increases the transverse RS of the upper surface and middle area of the plate’s thickness in the LAHW joint. After releasing the transverse restraint, the transverse RS of the LAHW joint decreases and restores to a level comparable to that of the welded joint under free condition (Case 2). A similar phenomenon can be found in Figure 23b, but the transverse RS in Case 3 (a) along Path L2 was only slightly higher than that in Case 2 and Case 3 (b). Figure 23a–c show that the transverse restraint increases the transverse RS by about 90 MPa on the upper surface, 60 MPa in the middle area of the plate’s thickness, and only 10 MPa on the bottom surface. This phenomenon is mainly related to uneven transverse shrinkage deformation in the plate’s thickness direction of the LAHW joint. Figure 24 compares the transverse shrinkage deformations along Paths L1, L2, and L3 as predicted in Case 2. The weld groove on the upper surface of the LAHW joint was larger than the weld groove on the bottom surface, resulting in the largest transverse shrinkage deformation on the upper surface (Path L1), followed by the middle area of the plate’s thickness (Path L3), and the smallest on the bottom surface (Path L2). The transverse shrinkage deformation of the LAHW joint under transverse restraint conditions (Case 3-a) is limited. The limited transverse shrinkage deformation on the upper surface was greater than that on the bottom surface. Therefore, the increment in the transverse RS induced by the transverse restraint on the upper surface was much higher than that on the bottom surface. A similar phenomenon was found in the transverse RS distribution along Path L4, as shown in Figure 23d.

## 5. Conclusions

Both experiments and numerical simulations were employed to investigate the welding RS distribution in the LAHW joint made of Q345 steel. Using ABAQUS software, the welding RS distribution in the LAHW joint was simulated by the sequentially coupled TMM FE method. The effects of SSPT and transverse restraint on the RS distributions were explored. The primary conclusions are summarized as follows:

(1) The similarity between the simulated molten pool morphology and the actual LAHW joint indicates the appropriateness of the cone–double ellipsoid hybrid heat source model in simulating the temperature field. The calculated phase fractions correspond to the microstructure and microhardness of the actual LAHW joint, indicating the reasonableness of taking SSPT into account in the material model. The good agreement between the experimentally measured data and the numerical simulation results of the RS indicates the correctness of the established sequentially coupled TMM FE method.

(2) SSPT has an important effect on the RS distribution in Q345 steel LAHW joints. Material models that do not consider the SSPT in numerical simulations will grossly overestimate the longitudinal RS in the FZ and HAZ of Q345 steel LAHW joints, as well as the transverse RS on the upper and bottom surfaces. The SSPT must be considered when simulating the welding RS distribution in LAHW joints of Q345 steel.

(3) Compared with the welding RS in the LAHW joint under free conditions, the transverse restraint significantly increased the transverse RS on the upper surface of the Q345 steel LAHW joint and had a slight effect on the transverse RS on the bottom surface and the longitudinal RS distribution. After releasing the transverse restraint, the longitudinal and transverse RS in the LAHW joint returned to a level comparable to that of the welded joint under free conditions.

## Figures and Tables

**Table 1:** Chemical compositions of Q345 steel and ER50-2 wire (mass fraction, %).

Material	C	Mn	Si	P	S	Cr	Cu	Fe
Q345	0.15	1.47	0.65	0.012	0.005	0.034	0.029	Bal.
ER50-2	0.09	1.20	0.66	0.016	0.02	0.01	0.11	Bal.

**Table 2:** Welding parameters employed in the LAHW joint.

Welding Current	Arc Voltage	Laser Power	Welding Speed	Filament Spacing
285 A	31 V	9 kw	2 m/min	2 mm

**Table 3:** Parameters for the hybrid heat source model.

*a_f_*_(mm)_	*a_r_*_(mm)_	*b*_(mm)_	*c*_(mm)_	*r_e_*_(mm)_	*r_i_*_(mm)_	*z_e_*_(mm)_	*z_i_*_(mm)_
3	5	3.5	4	0.7	1	6.5	−0.5

**Table 4:** Simulation cases.

Case	Solid-State Phase Transformation	Boundary Condition
Case 1	NO	Free condition
Case 2	YES	Free condition
Case 3	YES	Transverse restraint condition

## Data Availability

The original contributions presented in the study are included in the article, further inquiries can be directed to the corresponding authors.
